# Bioinformatics analysis of key genes and pathways in Hashimoto thyroiditis tissues

**DOI:** 10.1042/BSR20200759

**Published:** 2020-07-21

**Authors:** Long Zheng, Xiaojie Dou, Huijia Song, Pengwei Wang, Wei Qu, Xianghong Zheng

**Affiliations:** 1 Department of Nuclear Medicine, The Second Affiliated Hospital of Xi'an Jiaotong University, Xi’an 710004, Shaanxi, China; 2 Xi'an Jiaotong University Health Science Center, Xi’an 710061, Shaanxi, China

**Keywords:** bioinformatic analysis, GEO database, hashimoto thyroiditis, hub genes, key pathway

## Abstract

Hashimoto thyroiditis (HT) is one of the most common autoimmune diseases, and the incidence of HT continues to increase. Long-term, uncontrollable HT results in thyroid dysfunction and even increases carcinogenesis risks. Since the origin and development of HT involve many complex immune processes, there is no effective therapy for HT on a pathogenesis level. Although bioinformatics analysis has been utilized to seek key genes and pathways of thyroid cancer, only a few bioinformatics studies that focus on HT pathogenesis and mechanisms have been reported. In the present study, the Gene Expression Omnibus dataset (GSE29315) containing 6 HT and 8 thyroid physiological hyperplasia samples was downloaded, and differentially expressed gene (DEG) analysis, Gene Ontology analysis, Kyoto Encyclopedia of Genes and Genomes pathway enrichment analysis, protein–protein interaction analysis, and gene set enrichment analysis were performed. In total, 85 DEGs, containing 76 up-regulated and 9 down-regulated DEGS, were identified. The DEGs were mainly enriched in immune and inflammatory response, and the signaling pathways were involved in cytokine interaction and cytotoxicity. Moreover, ten hub genes were identified, and IFN-γ, IFN-α, IL6/JAK/STAT3, and inflammatory pathways may promote the origin and progression of HT. The present study indicated that exploring DEGs and pathways by bioinformatics analysis has important significance in understanding the molecular mechanisms of HT and providing potential targets for the prevention and treatment of HT.

## Introduction

Hashimoto thyroiditis (HT), also called autoimmune thyroiditis, is a lymphocyte-related chronic inflammation of the thyroid gland [1,2]. HT has been defined for more than a hundred years and has become the most common autoimmune disease [1]. According to previous reports, more than 20–30% of people suffer from HT, and the incidence of HT continues to increase [3,4].

The most common characteristic of HT is elevation of the following two thyroid autoimmune antibodies: anti-thyroperoxidase antibody (TPOAb) and anti-thyroglobulin antibody (TGAb). TPOAb or TGAb is detected in 95% of HT patients but rarely in healthy people [5]. Diagnosis of HT is mainly dependent on serum markers and sonography [6–8]. Although some patients are asymptomatic, long-term and uncontrolled HT may not only cause hypothyroidism or subclinical hypothyroidism in most cases, but also lead to hyperthyroidism in some cases [5,9]. However, there are other consequences of HT. Some studies have reported that HT causes severe cardiac effusion or cardiac tamponade independently or synergistically with other disease [10]. Excessively high titer of TPOAb and TGAb may cross blood–fetal barrier and lead to antenatal and neonatal disorders in pregnant patients [11].

Moreover, HT is considered to increase the risk of thyroid cancer. Silva et al. performed a prospective, cohort study containing 9851 consecutive patients with 21,397 nodules, and they found that the malignant risk of nodules in HT patients is 1.6 times higher than that in non-HT patients. Another study has shown that HT leads to a 3-fold increase in malignant risk compared with non-HT thyroid diseases and that the lymph node metastasis risk is four times greater than that in non-HT patients [12,13]. Therefore, blockade of HT at the source has important significance on decreasing prevalence and progression of thyroid cancer.

To date, daily, lifelong and oral levothyroxine administration is the main treatment for HT with permanent hypothyroidism or subclinical hypothyroidism. However, this is a therapy according to the symptoms rather than pathogenesis of HT [1]. Since the mechanisms of autoimmune diseases are complex and omnifarious, there are no available strategies for curing HT. Therefore, it is essential for clinicians to seek effective therapies to treat HT on a pathogenesis level and to prevent HT progression before the emergence of carcinogenic effects and other complications.

At present, bioinformatics analysis that combines medicine together with computational biology has become one of the hot fields of biomedical research. Large numbers of differential expression genes (DEGs) have been found, especially in cancer [14] or chronic diseases [15]. However, no previous HT-related bioinformatics analysis report has been published. In the present study, we searched the Gene Expression Omnibus (GEO) database and obtained the GSE29315 dataset, which contains gene expression data for HT and thyroid physiological hyperplasia (TPH), aiming to explore the hub genes and relevant signaling pathways functioning in HT.

## Materials and methods

### Dataset selection

The search words “Hashimoto thyroiditis” OR “autoimmune thyroiditis” AND “human” AND “Expression profiling by array” were applied for dataset retrieval. Most of the current databases, such as TCGA and Oncomine, mainly provide high-throughput microarray data on cancer-related research, and few databases, such as GEO database, provide data on nontumor diseases. Therefore, only one dataset (GSE29315) belonging to GEO was selected to perform the following bioinformatics analysis. Since the GSE29315 dataset contains 8 TPH samples and 6 HT samples without normal samples, we selected TPH as the control group in the present study.

### Identification of DEGs

The expression matrices of the GSE29315 and GPL8300 platform files were downloaded from the GEO website and were transformed into expression matrix data and grouping data by using manual sorting and Perl language software. R language was utilized for standardization of all expression data. The analysis of DEGs was performed using limma R package. Genes with an expression fold change >4 and *P*<0.05 were regarded as DEGs. The volcano and heatmap plots were generated by R packages.

### Gene Ontology (GO) and Kyoto Encyclopedia of Genes and Genomes (KEGG) pathway enrichment analyses of DEGs

GO and KEGG enrichment analyses were executed using Database for Annotation, Visualization and Integrated Discovery (DAVID) (https://david.ncifcrf.gov). All DEG symbols were input as a list into the DAVID website, and the “homo sapiens” sample type was selected. The results of GO enrichment and KEGG pathway analyses were provided by bioinformatics tools in the website. There are three main processes in GO analysis as follows: biological processes, molecular functions, and cellular components. The main pathways of KEGG included metabolism, genetic information processing, environment information-related processes, cell physiological processes, and drug research. Bar plots were drawn using R commands based on data acquired from GO and KEGG enrichment results.

### Protein–protein interaction network analysis

Protein–protein interaction (PPI) analysis was performed using the searching tool for retrieval interacting genes (STRING) 10.0 (https://string-db.org/), which is software used to explore interactions among proteins. All the symbols of DEGs were input, and the “homo sapiens” sample type was selected. The primary data of interactions among proteins were downloaded. Cytoscape is an excellent application for network visualization of proteins and was used to generate the PPI network plot constructed by nodes and edges. The top 10 hub genes were predicted by Cytohubba, which is a plugin of Cytoscape to screen hub genes from numerous candidates. The ranks of the 10 hub genes in the network were determined by the value of “Degree”, which is an algorithm provided by Cytohubba. Because the degree value of a gene is directly related to its importance, genes with a high degree value are more likely to be hub genes.

### Gene set enrichment analysis (GSEA)

GSEA is a microarray data analysis tool that is used to analyze biological information. In the present study, all DEGs were used for GSEA analysis. Pathways with *P*<0.05 were considered as significant signaling pathways.

## Results

### Identification of DEGs between HT and TPH

**Figure 1:**
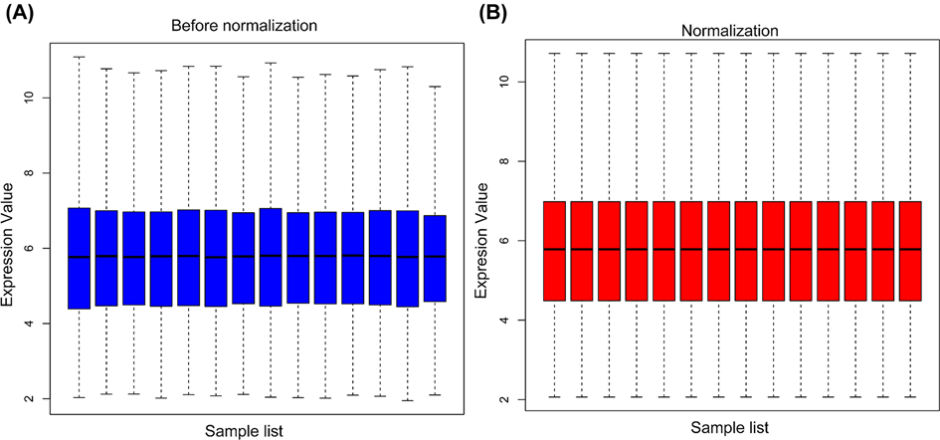
Standardization of gene expression data in samples (**A**) Before standardization; (**B**) After standardization. The blue bar represents the data before normalization, and the red bar represents the data after normalization.

**Figure 2:**
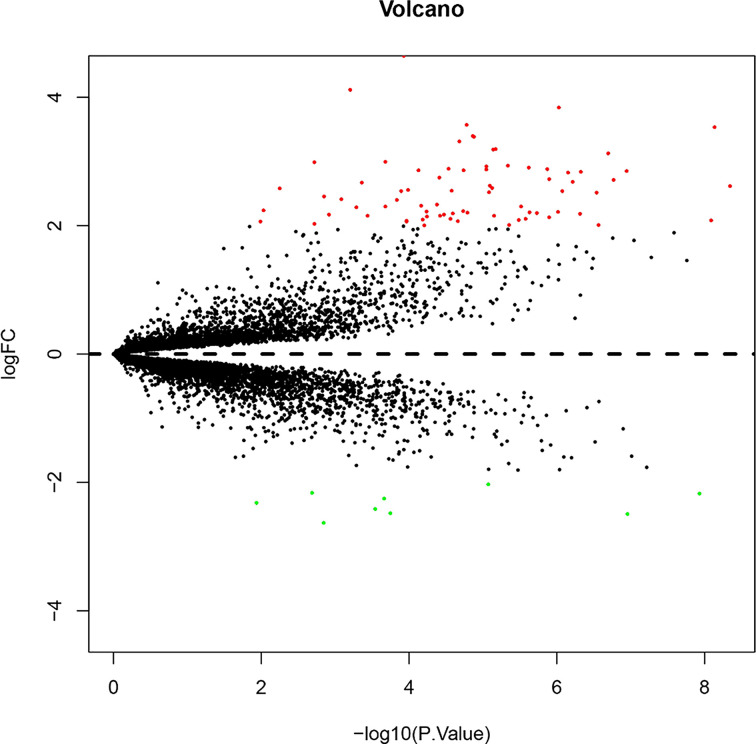
Volcano plot of DEGs between HT and TPH Red points represent up-regulated genes, and green points represent down-regulated genes. Genes with no significant difference are shown in black. The differences are set as |logFC|>2 and *P*<0.05.

**Figure 3:**
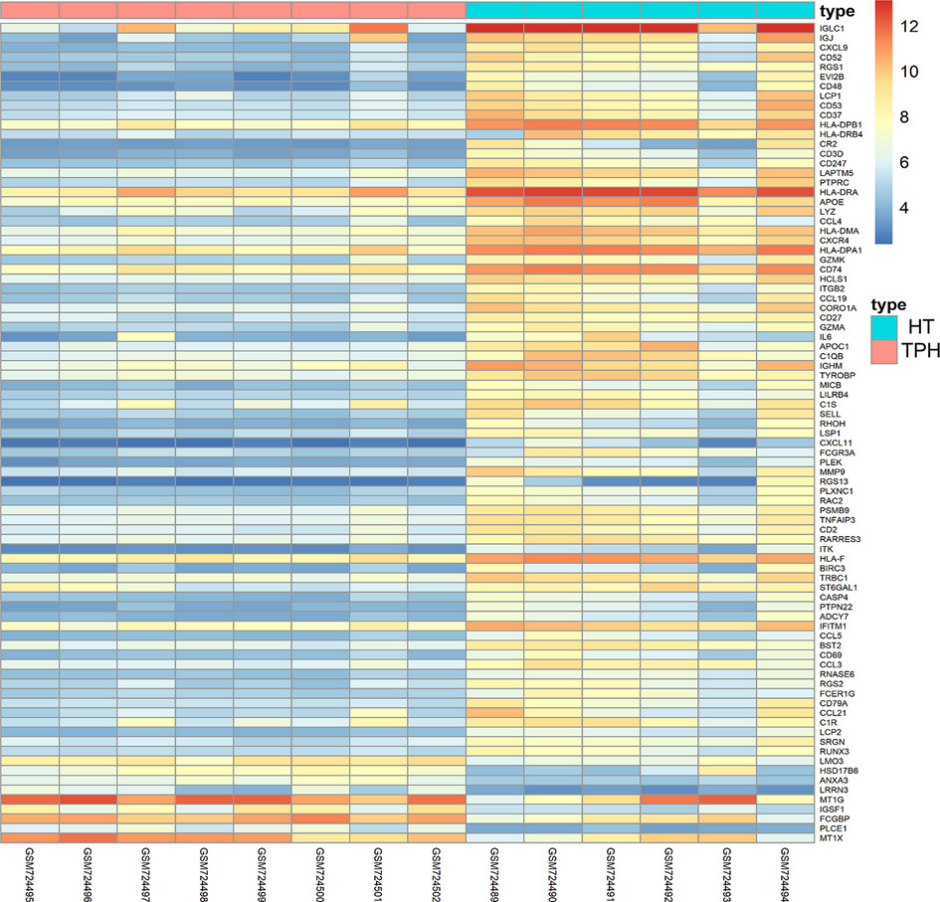
Heatmap of the DEGs Up-regulated genes are shown in red, while down-regulated genes are shown in blue.

The expression data of the 8 TPH samples and 6 HT samples in GSE29315 were standardized (Figure 1). In total, 85 DEGs, containing 76 up-regulated and 9 down-regulated genes, were identified by limma R package, and the replicate gene symbols were deleted. A volcano plot was used to show the cluster of DEGs (Figure 2), and the heatmap plot was constructed by the pheatmap R package to visually show all DEGS in the TPH and HT samples (Figure 3). Among the identified DEGs, the top 10 up-regulated genes were IGLC1, IGJ, CXCL9, CD52, RGS1, EVI2B, CD48, LCP1, CD53, and CD37. In addition, the 9 down-regulated genes were LMO3, HSD17B6, ANXA3, LRRN3, MT1G, IGSF1, FCGBP, PLCE1, and MT1X. The detailed information for all DEGs is shown in Supplementary Tables S1 and 2.

### Gene and pathway enrichment analysis

**Figure 4:**
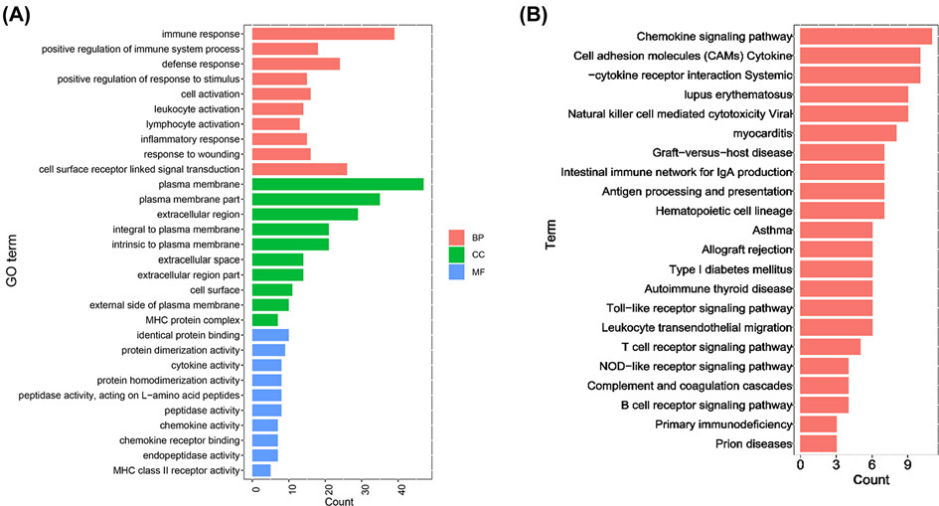
GO and KEGG enrichment analyses of DEGs in HT (**A**) GO analysis divided DEGs into three groups as follows: biological processes (red bars), cell components (green bars), and molecular functions (blue bars). (**B**) Bar plot of KEGG pathway enrichment analysis.

The GO terms of the DEGs were divided into three groups as follows: biological process, molecular functions, and cellular components (Figure 4A). The DEGs in our study were mainly enriched in biologic processes and molecular functions. In biologic process, these identified genes were mainly enriched in immune response, defense response, cell surface receptor-linked signaling transduction, inflammatory response, leukocyte activation, and lymphocyte activation. The genes were also enriched in plasma membrane, extracellular region, and extracellular space in cellular components. Moreover, we also submitted the DEGs to KEGG pathway enrichment analysis. As shown in Figure 4B, the involved pathways were mainly enriched in chemokine, cytokine-to-cytokine interaction, cell adhesion molecules, natural killer cell-mediated cytotoxicity, antigen processing, and antigen presentation. In addition, the KEGG results indicated that the DEGs of HT may also function in other immune-related diseases, such as asthma, graft-versus-host disease, Type 1 diabetes mellitus, and viral myocarditis.

### Investigation of HT hub genes by PPI network analysis

**Figure 5:**
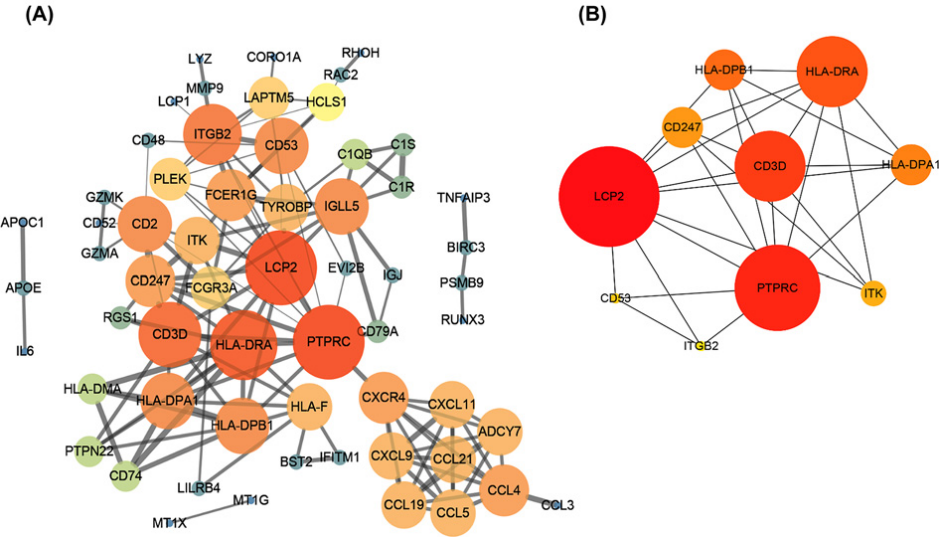
PPI network and hub genes in HT (**A**) PPI network. (**B**) Hub genes and interaction in HT. Circles represent genes, and lines represent interaction among DEGs.

The identified DEGs were submitted into the STRING database to acquire PPI data. We applied Cytoscape 3.6.1 to construct the PPI network. After removal of isolated nodes, a PPI network of DEGs was generated (Figure 5A). The top 10 hub genes were identified by Cytohubba, a plugin of Cytoscape software (Figure 5B). All hub genes were up-regulated DEGs, including LCP2, PTPRC, HLA-DRA, CD3D, HLA-DPB1, HLA-DPA1, CD247, ITK, CD53 and ITGB2.

### Identification of significant pathways of HT by GSEA

**Figure 6:**
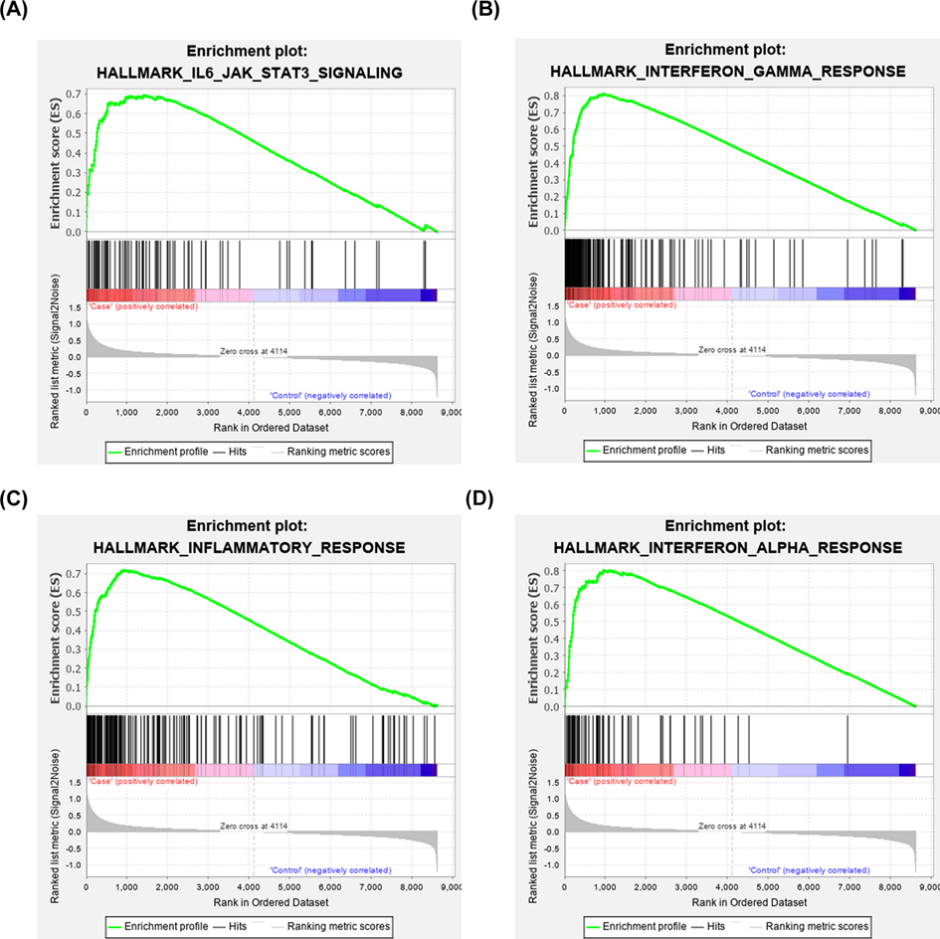
GSEA of enriched pathways in HT (**A**) IL-6/JAK/STAT3 pathway; (**B**) IFN-γ response pathway; (**C**) inflammatory response pathway; (**D**) IFN-α response pathway.

We performed GSEA to further explore the key pathways of HT. With *P*<0.05 as the inclusion criteria, we confirmed that four candidate pathways (IL6/JAK/STAT3, IFN-γ, IFN-α, and inflammatory pathways) may be related to the origin and development of HT (Table 1 and Figure 6).

**Table 1:** Results of GSEA analysis

Pathway	*P* value	Gene number
IL6/JAK/STAT3	0.045082	77
Inflammatory response	0.038306	177
Interferon-α response	0.022403	65

## Discussion

In the present study, 85 DEGs of HT were identified, including 76 up-regulated and 9 down-regulated genes. The DEGs are mainly enriched in biological processes and cellular components. Inflammatory response and immune receptor-related signaling were the enriched pathways of the DEGs. LCP2, PTPRC, and eight other genes were identified as hub genes that may affect HT origin and development, and four pathways (IL-6/JAK/STAT3 pathway, IFN-α signaling, IFN-γ signaling, and inflammatory response) were identified to be potential key pathways in HT.

The incidence of HT continues to increase. Studies on the pathogenesis and diagnostic biomarkers are essential for the early diagnosis and treatment of HT. HT is an autoimmune disease, and its specific thyroid antibodies are generated by disorder of T and B cells [16,17]. Because the origin and development of HT involve many immune molecules and signaling pathways, it is difficult to completely understand the pathogenesis of HT. Our present findings may provide ideas for the further HT studies.

By PPI network analysis, we identified the top 10 hub genes that may affect the origin or development of HT. According to the interaction degree score calculated by Cytohubba, LCP2, PTPRC, HLA-DRA, and CD3D may play important roles. Lymphocyte cytosolic protein 2 (LCP2) is located on Chromosome 5q33.1 and encodes a 76 kD leukocyte protein, which enhances the IL-2 promoter and functions in T-cell activation [18]. However, other functions of LCP2 are not well known. Protein tyrosine phosphatase receptor type C (PTPRC) is a member of the PTP family and promotes autoimmune diseases. A previous study has found that PTPRC activates JAK and STAT proteins by suppressing JAK kinase, leading to autoimmune disorder in systemic lupus erythematosus [19]. Lee et al. performed a meta-analysis using eight studies with a total of 3058 patients, and they demonstrated that PTPRC polymorphisms result in poor response to anti-TNF therapy in treatment of rheumatoid arthritis [20]. CD3D, a T-cell receptor, was first reported in 1989 [21]. Aparicio et al. found CD3D polymorphisms in type 1 diabetes mellitus and indicated it as a potential gene marker [22]. Leaky mutation of CD3D may lead to a T-cell immune defect, but the consequence of its upregulation has not been reported [23]. Thus far, no study has reported the function of the above three genes (LCP2, PTPRC and CD3D) in HT. Human leukocyte antigen (HLA) class II genes and their alleles are considered as the pivotal factors of HT. HLA and its alleles may confer the strongest susceptibility to autoimmune poly-glandular syndrome 3 variant (APS3v), which is associated with the occurrence of HT [24,25]. In the present study, we identified not only classic HT susceptibility genes, but also several novel unreported gene markers, thereby supporting the credibility of our bioinformatics analysis.

Apart from the hub genes, the present study also identified several key pathways in HT. According to the GSEA, four pathways (IL-6/JAK/STAT3, IFN-α, IFN-γ, and inflammatory response) were identified in HT. The IL-6/JAK/STAT3 pathway plays a pivotal role in immune regulation and secretion of cytokines, which affects the generation or development of HT. Overactivation of STAT3 may enhance susceptibility and elevate thyroid autoantibody titers in HT patients [26–28]. Some cases have reported that administration of IFN-α or IFN-γ to treat other diseases leads to autoimmune thyroid disorders [29,30]. Excessive interferon levels trigger HT through various immune response processes, such as lysosomal-dependent degradation of thyroglobulin [31]. In the future, drugs or therapeutic strategies targeting these pathways may effectively prevent the occurrence or progression of HT.

High-throughput sequencing analysis has been widely applied to seek potential targets in human cancer. Moreover, this technology has also been used in nontumor diseases, such as diabetes mellitus [32], coronary artery disease, and ischemic cardiomyopathy [15]. Until now, no relevant study identifying the hub genes and pathways of HT has been reported. The results of our bioinformatics analysis will be helpful in future studies on HT.

There are some limitations in the present study. First, only one dataset containing high-throughput sequencing data for HT was selected from the GEO database. In addition, we did not retrieve high-throughput data for HT in other databases because most of the current databases, such as TCGA and Oncomine, mainly provide high-throughput microarray data on cancer-related research and few databases provide data on nontumor diseases, such as coronary artery disease, and diabetes mellitus. Thus, additional microarray and high-throughput sequencing studies needed to study HT. Second, we selected TPH as the control group because the selected GSE29315 database did not include microarray data for normal thyroid tissues. Considering the potential analysis bias, further molecular biology experiments to verify the function of hub genes on a cell or specimen level will be performed in our subsequent work.

## Conclusion

In conclusion, we identified 85 DEGs based on the GSE29315 dataset downloaded from the GEO database. Through GO and KEGG enrichment analyses, we found that the DEGs were mainly enriched in biological processes and cellular components. We obtained 10 hub genes and 4 key pathways that may be strongly associated with HT. In the future, additional datasets and further experiments on a cell or specimen level are needed to validate our findings in the origin and development of HT.

## Supplementary Material

Supplementary Tables S1-S2

## Data Availability

The raw data contained expression matrix and GPL information from the GSE29315 dataset downloaded from the GEO database. The relevant codes are available upon request.

## Abbreviations

DEG: differentially expressed gene
GEO: gene expression omnibus
GO: gene ontology
GSEA: gene set enrichment analysis
HT: Hashimoto thyroiditis
KEGG: Kyotoe ncyclopedia of genes and genomics
PPI: protein–protein interaction
TPH: thyroid physiological hyperplasia
