# Supplementary material for: Bioinformatics analysis of key genes and pathways in Hashimoto thyroiditis tissues
Source: Biosci Rep. 2020 Jul 21;40(7):BSR20200759. doi: 10.1042/BSR20200759 (PMC7374273; doi:10.1042/BSR20200759)
Supplement: Supplementary Tables S1-S2 [file BSR-2020-0759_supp.pdf]

Supplementary Table 1. DEGs upregulated in HT

Gene symbols	LogFC	AverageExpression	*P*-value
IGLC1	4.6450596	9.9904051	0.0001182
IGJ	4.1157793	6.9338384	0.000627
CXCL9	3.8388564	5.915452	9.39E-07
CD52	3.5711165	6.1763976	1.67E-05
RGS1	3.5363718	6.0769872	7.31E-09
EVI2B	3.3977522	4.8862804	1.38E-05
CD48	3.3834423	4.7612914	1.33E-05
LCP1	3.3115106	6.6877506	2.08E-05
CD53	3.1942522	6.8369609	6.72E-06
CD37	3.1841628	7.0042121	7.26E-06
HLA-DPB1	3.1288995	9.234291	2.02E-07
HLA-DRB4	2.9957583	6.694206	0.0002094
CR2	2.9902755	4.7387722	0.0019165
CD3D	2.9370359	5.0134721	4.60E-06
CD247	2.9192602	5.8213114	9.00E-06
LAPTM5	2.9034381	7.8658074	2.40E-06
PTPRC	2.8889813	6.5418187	2.92E-05
HLA-DRA	2.8814965	10.711587	1.34E-06
APOE	2.8752205	8.8323933	9.01E-06
LYZ	2.8636476	7.5660133	7.45E-05
CCL4	2.8595879	6.1137385	1.83E-05
HLA-DMA	2.8485622	8.385643	1.14E-07
CXCR4	2.8361486	7.7597386	4.73E-07
HLA-DPA1	2.8260451	9.6760793	7.02E-07
GZMK	2.7474446	6.2170755	3.88E-05
CD74	2.7250783	9.4783822	1.27E-06
HCLS1	2.7121311	7.4869909	1.71E-07
ITGB2	2.6816059	5.7755055	6.08E-07
CCL19	2.6684394	5.9557595	0.0004356
CORO1A	2.6209167	7.4640226	8.08E-06
CD27	2.6133058	6.72791	4.52E-09
GZMA	2.5828093	6.255933	7.48E-06
IL6	2.5769565	5.290483	0.0056276
APOC1	2.553726	7.1693962	0.0001036
C1QB	2.5413433	7.4137841	2.65E-05
IGHM	2.5405204	8.2209914	0.0001276
TYROBP	2.536903	7.7714925	8.44E-07
MICB	2.5169696	5.3218451	8.30E-06
LILRB4	2.5166348	6.026356	2.88E-07
C1S	2.4543733	7.4238569	0.0014145
SELL	2.4118598	5.7175381	0.0008284
RHOH	2.4020333	4.8156153	0.0001471
LSP1	2.3274742	5.981778	4.20E-05
CXCL11	2.3075595	3.5570676	6.86E-05
FCGR3A	2.2989995	5.8755628	0.0002087
PLEK	2.2947957	4.5895165	3.04E-06
MMP9	2.2829609	6.7217016	0.0005173
RGS13	2.2386741	3.5089505	0.0092951
PLXNC1	2.2273933	5.5308344	1.85E-05
RAC2	2.2211546	5.4664236	5.82E-05
PSMB9	2.213296	7.2647999	9.65E-07
TNFAIP3	2.2086899	7.2146217	2.34E-06
CD2	2.2036707	6.9034569	1.63E-05

RARRES3	2.1963807	7.3521843	1.86E-06
ITK	2.1910698	4.1222686	2.55E-05
HLA-F	2.1824097	9.4858579	4.84E-07
BIRC3	2.1729033	4.9048128	0.0012139
TRBC1	2.1725406	7.8047136	3.36E-05
ST6GAL1	2.1566643	7.3319627	0.0003654
CASP4	2.1530751	5.2220154	7.06E-06
PTPN22	2.1519749	4.7847395	3.80E-05
ADCY7	2.141864	4.9218457	5.74E-05
IFITM1	2.1293099	8.6532159	1.28E-06
CCL5	2.1050406	5.120152	2.76E-05
BST2	2.1033596	7.2185822	2.66E-06
CD69	2.094661	5.2264458	6.55E-05
CCL3	2.0877234	7.0489096	3.28E-06
RNASE6	2.0806245	5.3576155	8.18E-09
RGS2	2.0782212	6.0053551	0.0001077
FCER1G	2.0724565	5.7229898	2.18E-05
CD79A	2.0634681	6.3369802	0.0001078
CCL21	2.0628885	6.3812505	0.0102551
C1R	2.0305671	7.1748136	0.0019107
LCP2	2.0107815	4.9959251	2.73E-07
SRGN	2.006958	6.4013788	4.42E-06
RUNX3	2.0057192	6.0856356	6.20E-05

Supplementary Table 2. DEGs downregulated in HT

Gene symbols	LogFC	Average Expression	*P*-value
MT1X	2.633672	9.4037443	0.001433
PLCE1	2.493008	5.2171852	1.10E-07
FCGBP	2.479739	9.444249	0.0001783
IGSF1	2.414623	6.8030208	0.0002874
MT1G	2.318752	10.480028	0.0116428
LRRN3	2.25048	4.7014886	0.0002183
ANXA3	2.175752	5.921536	1.18E-08
HSD17B6	2.165722	6.5737528	0.0020567
LMO3	2.033753	7.8959711	8.46E-06
